# A Case of Inverted Uterus

**Published:** 1871-04

**Authors:** George Rightmire

**Affiliations:** Jacksonville, N. Y.


ART. III.—*A Case of Inverted Uterus.* By GEORGE RIGHTMIRE. Jacksonville, N. Y., March 17th, 1871.

On the morning of Jan. 26th, 1871, Mrs. R., of Enfield, Tompkins county, N. Y., was confined, by Dr. H., with her first child. The labor was somewhat tedious, but otherwise the same as is usually met with. The child was large, and the pelvis small, so that considerable force was used to deliver her.

Immediately after delivery there was hemorrhage, and fainting accompanying it. The Doctor, becoming alarmed, attempted to extract the placenta by making strong traction on the cord. Not succeeding, he introduced his hand into the vagina, and found a tumor in the upper part coming down from the uterus, (which he supposed to be some fibrous tumor,) with the placenta attached to it. He detached the placenta as well as he could, and took it away in pieces. Flooding and fainting then ceased.*

On the 30th of January, Dr. CHASE was called in consultation, and, just before he arrived, Dr. H. drew off nearly a gallon of urine

*The above is Dr. H.'s own statement.

which had been neglected since delivery. Dr. C., on making an examination, found the labia majora much swollen, and perinæum lacerated. On introducing his hand into the vagina, he found the uterus completely inverted, indurated and congested.

The patient was then placed under the influence of chloroform, and an attempt made to return the uterus, which failed. After this the patient was placed upon the use of anodynes, and low diet, to prevent inflammation. She continued in about the same condition, lying quietly on her back all the while, without any hemorrhage, until the 27th of February, 1871, when Dr. JAMES P. WHITE, of Buffalo, (whose success in such operations is well known throughout New York and adjoining States,) having been called, examined the parts and confirmed the diagnosis of Dr. C., viz., that it was a case of Complete Inversion of the Uterus. He then administered chloroform and ether, and succeeded in repositing the uterus in twenty three minutes.

The patient vomited during the remainder of the day and night, but ceased the next morning. Since that time she has been gradu ally convalescing, and is now able to sit up part of the time.

